# A Comparison of Muscle Activity in Concentric and Counter Movement Maximum Bench Press

**DOI:** 10.2478/hukin-2013-0046

**Published:** 2013-10-08

**Authors:** Roland van den Tillaar, Gertjan Ettema

**Affiliations:** 1 Department of Teacher Education of Nord Trøndelag University College, Levanger Norway.; 2 Department of human Movement Science, Faculty of Social Sciences and Technology Management, Norwegian University of Science and Technology (NTNU), Trondheim, Norway.

**Keywords:** kinematics, EMG, muscle, potentiation, bench press

## Abstract

The purpose of this study was to compare the kinematics and muscle activation patterns of regular free-weight bench press (counter movement) with pure concentric lifts in the ascending phase of a successful one repetition maximum (1-RM) attempt in the bench press. Our aim was to evaluate if diminishing potentiation could be the cause of the sticking region. Since diminishing potentiation cannot occur in pure concentric lifts, the occurrence of a sticking region in this type of muscle actions would support the hypothesis that the sticking region is due to a poor mechanical position. Eleven male participants (age 21.9 ± 1.7 yrs, body mass 80.7 ± 10.9 kg, body height 1.79 ± 0.07 m) conducted 1-RM lifts in counter movement and in pure concentric bench presses in which kinematics and EMG activity were measured. In both conditions, a sticking region occurred. However, the start of the sticking region was different between the two bench presses. In addition, in four of six muscles, the muscle activity was higher in the counter movement bench press compared to the concentric one. Considering the findings of the muscle activity of six muscles during the maximal lifts it was concluded that the diminishing effect of force potentiation, which occurs in the counter movement bench press, in combination with a delayed muscle activation unlikely explains the existence of the sticking region in a 1-RM bench press. Most likely, the sticking region is the result of a poor mechanical force position.

## Introduction

The bench press is one of the most popular exercises used in strength training for the upper body. A successful bench press lift is performed when the barbell is first lowered to the chest and then moved to a fully extended position again. Several studies have investigated the kinematics in bench pressing and have shown that there is a sticking region during maximal lifts (Madsen and McLaughlin, 1984; Newton et al., 1997). In this region, the pushing force is less than gravity on the barbell, leading to a deceleration of the barbell. It is defined as the movement region from peak velocity (vmax) to the first local minimum velocity (vmin) of the upward barbell movement (Lander et al., 1985; Elliot et al., 1989; van den Tillaar and Ettema, 2010).

The reason for the existence of this sticking region is unclear. Elliott et al. (1989) and Madsen and McLaughlin (1984) hypothesized that during the sticking region a poor mechanical force position occurs in which the lengths and mechanical advantages of the muscles involved are such that their capacity to exert torque is reduced. Van den Tillaar and Ettema (2010) found that the muscle activity of only the agonistic major pectoralis muscles and the anterior part of the deltoid muscles increased from the sticking to the post-sticking region during the upward movement. They proposed that the start of a sticking region occurs, not because of a lack of strength, but due to diminishing of enhanced force (i.e., potentiation induced by the immediately preceding eccentric contraction) at the start of the concentric movement. When this strength capacity is diminishing, a delayed neural reaction must occur (Walshe et al., 1998) enhancing the muscle activity level so that the resultant force matches the demands of the attempt. Thus, the delay in neural activity increase would be the cause of the sticky region, whereas the increase itself results in the overcoming of the sticking region (Van den Tillaar and Ettema, 2010).

In the above mentioned studies the bench press was performed with a downward and subsequent upward movement (muscular stretch-shortening cycle), which can cause potentiation and its diminishment over time and thereby the occurrence of the sticking region. Since in pure concentric lifts these mechanisms cannot play a significant role, these lifts can be used to test whether the sticking region is caused by potentiation and an accompanying delayed neural reaction (Van den Tillaar and Ettema, 2010), or if it is due to a poor mechanical force position (Madsen and McLaughlin, 1984; Elliott et al., 1989). Wilson et al. (1991) showed that subjects can lift around 14% more with a counter movement bench press than with a pure concentric lift. Furthermore, they found that the force output only during the first 200ms was lower when performing pure concentric bench presses. However, they only analysed the first 0.5s of the ascending part of the lift. Moreover, they did not explain the reason for these differences (and did not perform any EMG measurements of the involved muscles). In squatting, Walshe et al. (1998) also found differences in force output only during the first 300ms of the ascending part. They showed that there were no differences in muscle activity between the two conditions. Walshe et al. (1998) concluded that the difference was caused by the attainment of a higher active state before the start of the upwards movement. It was also hypothesized that the contractile element potentiation was a significant contributor to the performance of the counter movement performance.

Therefore the aim of the present study was two folded: firstly to compare the kinematics and muscle activation patterns of the regular free-weight bench press (counter movement) with pure concentric lifts and secondly, to test if diminishing potentiation could be the cause of the sticking region. Since diminishing potentiation cannot occur in pure concentric lifts, the occurrence of a sticking region in this type of muscle actions would support the hypothesis that the sticking region is due to a poor mechanical position. Furthermore, similarity of activation patterns in the pure concentric 1RM lift and regular 1RM bench press would support the poor-mechanical-position hypothesis.

## Material and Methods

### Participants

Eleven male participants (age 21.9 ± 1.7 yrs, body mass 80.7 ± 10.9 kg, body height 1.79 ± 0.07 m) with at least one year of bench press training experience (bench press training at least once or twice per week) participated in this study. The participants did not perform any additional resistance training exercises that were targeted at the chest, shoulders and upper arm muscle groups 72 hours prior to the test. The study complied with the approval of the local committee for medical research ethics and the current ethical standards in sports and exercise research.

### Procedures

A repeated measures design was used to compare the kinematics and muscle activation patterns of the regular free-weight bench press (counter movement) with pure concentric lifts in male subjects. To test the hypothesis about diminishing potentiating and delayed muscle activation, kinematics and EMG measurements were investigated for three regions (pre-, sticking and post-sticking region) in the concentric part under both 1-RM conditions.

The participants followed the same warm up protocol as used by Saeterbakken et al. (2011). They started with an own selected regular procedure to warm up the shoulders and arms followed by four warm-up sets: 1) twenty repetitions at 30% of 1-RM, 2) twelve repetitions at 50% of 1-RM, 3) six repetitions at 70% of 1-RM and 4) one repetition at 85% of 1-RM. The percentage of the 1-RM was estimated based on the self-reported 1-RM of the participants. The self-reported 1RM was set according to the information given by the participants on maximal lifts performed in the previous six months. The rest periods between the sets were around 3 to 5 minutes to avoid possible fatigue. When the self-reported 1-RM was successful, an attempt with an additional 2.5–5 kg was performed. When the initial attempt was unsuccessful the weight was decreased by 2.5–5 kg. A total of two to three attempts were performed by each participant. Only the attempt with the highest weight that was lifted successfully was used for further analysis.

Firstly the participants performed a traditional bench press (descending and ascending the barbell). No marked pause between descending and ascending the barbell was necessary. However, the participants were not permitted to “bounce” the barbell off the chest and were not allowed to raise the lower back from the bench. The position of the hands on the barbell was individually selected, but the forefinger had to be inside of the 91 cm mark on a standard Olympic bar. The positioning of the hands was taped to be sure that the distance between the hands was identical for each participant during the whole experiment. During each attempt two experienced spotters helped to lift the barbell from the stands and they slipped the barbell when the participant had full control over the barbell.

After accomplishing the 1-RM in this counter movement bench press (CM) the participant established the 1-RM in a pure concentric bench press (CONC). In the pure concentric bench presses the participants started to lift from the same lowest barbell height as in the lowest barbell position of the counter movement bench presses. Between the counter movement and concentric bench press the participants rested for around 10 minutes to avoid fatigue. In the concentric condition the barbell was lying on two stands on the lowest position touching the chest. The participants had to push the barbell up as quickly as possible on a signal given by the researcher. Two spotters were standing beside the barbell for security. From pilot testing it was found that on average a participant could lift around 20 kg less in the pure concentric bench press. Thus after evaluation of 1-RM in the counter movement bench press the weight was decreased by 20 kg when testing the 1-RM in the concentric condition. Also in this case, two to three attempts were made to establish the 1-RM in the concentric condition. During each attempt the participants were verbally supported to conduct the lift with maximal effort.

A linear encoder (Ergotest Technology AS, Langesund, Norway) connected to the barbell or dumbbells measured the vertical position and lifting time of the barbell during the attempts with a 0.075mm resolution and the pulses counts of 10 millisecond intervals (Arnason et al., 2004). The vertical displacement was measured in relation to the lowest point of the barbell (zero distance). Velocity, force and acceleration of the barbell were calculated using respectively a five point differential and a double differential filter. Also the total impulse defined as the integral of the force during the ascending phase was calculated with software Musclelab V8.13 (Ergotest Technology AS, Langesund, Norway). The linear encoder was synchronized with the EMG recordings using a Musclelab 3010e and analyzed by software V8.13 (Ergotest Technology AS, Langesund, Norway).

Before the 1-RM experimental test, the skin was prepared (shaved, washed with alcohol, abraded) for placement of gel coated surface EMG electrodes. Electrodes (11 mm contact diameter) were placed on the dominant side of the body on the belly of the muscle in the presumed direction of the underlying muscle fibers with a center-to-center distance of 2.0 cm according to the recommendations by SENIAM (Hermens et al., 2000). Self-adhesive electrodes (Dri-Stick Silver circular sEMG Electrodes AE-131, NeuroDyne Medical, USA) were positioned on the belly of the pectoralis major (sternocostal head), the anterior and medial deltoid, the lateral and medial triceps brachii and biceps brachii (short head) (Saeterbakken et al., 2011). To minimize noise induced from external sources, the EMG signal was amplified and filtered using a preamplifier located as near the pickup point as possible. The EMG signals were sampled at a rate of 1000 Hz. Signals were band pass filtered with a cut off frequency of 8 Hz and 450 Hz, after which the root-mean-square (RMS) was calculated. The RMS-converted signal was re-sampled at a rate of 100 Hz using a 16-bit A/D converter with a common mode rejection rate of 106 dB. The stored data were analyzed using commercial software (Musclelab V8.10, Ergotest Technology AS, Langesund, Norway).

**Figure 1:**
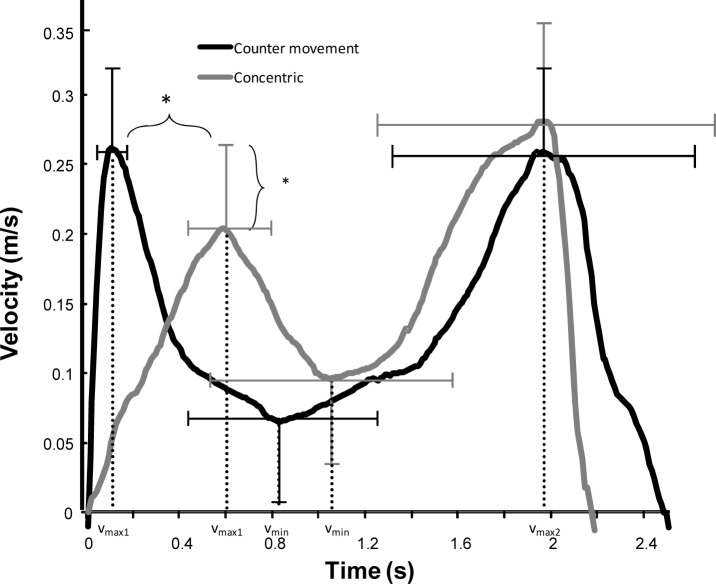
The upwards barbell movement velocity during a counter movement and concentric free-weight 1RM bench press with standard deviation in which the different events are shown: first peak velocity (v_max1_), second peak velocity (v_max2_) and the minimal velocity (v_min_) with the standard deviation averaged over the subjects.* indicates a significant difference at p ≤ *0.05 between the two conditions*

To locate possible differences in muscle activity during the 1-RM bench press movement, the average root mean square (RMS) was calculated for each of three regions. The first region was from the lowest barbell point (v_0_) until the maximal barbell velocity (v_max1_): the pre-sticking region. The second region was from the maximal barbell velocity until the first located lowest barbell velocity (v_min_): the sticking region. The last period, the post-sticking region, started at v_min_ to the second maximal barbell peak velocity (v_max2_), which is also called the strength region (Figure 1) (Lander et al., 1985).

### Statistical Analysis

To assess differences in neuromuscular activity in the three regions during the traditional and pure concentric condition, a repeated 2-way (condition: CM vs. CONC) × 3 (region: pre-sticking, sticking and post-sticking) analysis of variance (ANOVA) design was used. Bonferroni post hoc analyses were conducted to locate differences. For the other kinematics (time, position, velocity force, impulse, and acceleration) a paired t-test was conducted between the two conditions. All results are presented as mean ± SD. In case the sphericity assumption was violated, the Greenhouse-Geisser adjustments of the p-values were reported. The criterion level for significance was set at p < 0.05. Statistical analysis was performed in SPSS version 18.0 (SPSS, Inc., Chicago, IL).

## Results

The average weight that successfully was lifted in the counter movement bench press by the participants at 1-RM was 121.4 kg ± 29 kg, while the participants lifted less in the pure concentric condition (102.7 kg ± 21 kg; p<0.001).

**Figure 2:**
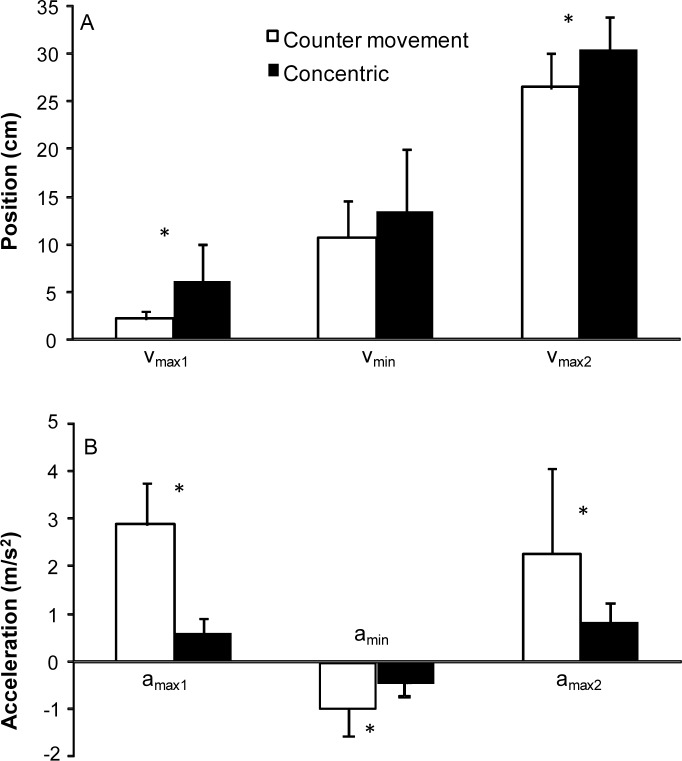
Position and acceleration of the first (v_max1_) and second peak velocity (v_max2_) and the minimal velocity (v_min_) during the counter movement and concentric bench press. * indicates a significant difference at p ≤ *0.05 between the two conditions*

No significant difference (*p* = 0.20) in total upwards lifting time (CM: 2.8±1.1 s vs. CONC: 3.3±1.4 s) was found between the two conditions (Figure 1). However, the time of the first peak velocity occurred later in the CONC condition (0.6±0.2 s vs. 0.13±0.04 s; *p* < 0.001; Figure 1), with no significant differences in the time of the minimal and second peak velocity between the two conditions (Figure 1). The position of the barbell at the first and second peak velocity was also significantly different (*p* < 0.006; Figure 2A), i.e. the distance between the chest and the barbell was higher at these two positions in the CONC condition. However, the first peak velocity was higher in the CM condition compared to the CONC condition (Figure 1; *p* = 0.014). The acceleration during the pre-sticking (*p* < 0.001) and post-sticking region (*p* = 0.033) were higher in the CM condition as shown by the steeper increase of velocity in Figure 1. In the sticking region the deceleration was also higher (*p* = 0.007) in the CM bench press condition compared to the CONC condition (Figure 2B).

The total impulse during the 1-RM attempts with the two conditions was approximately the same (CM: 3402±2204 Ns vs. CONC: 3414±1833 Ns)

### Muscle activity between the two conditions

**Figure 3:**
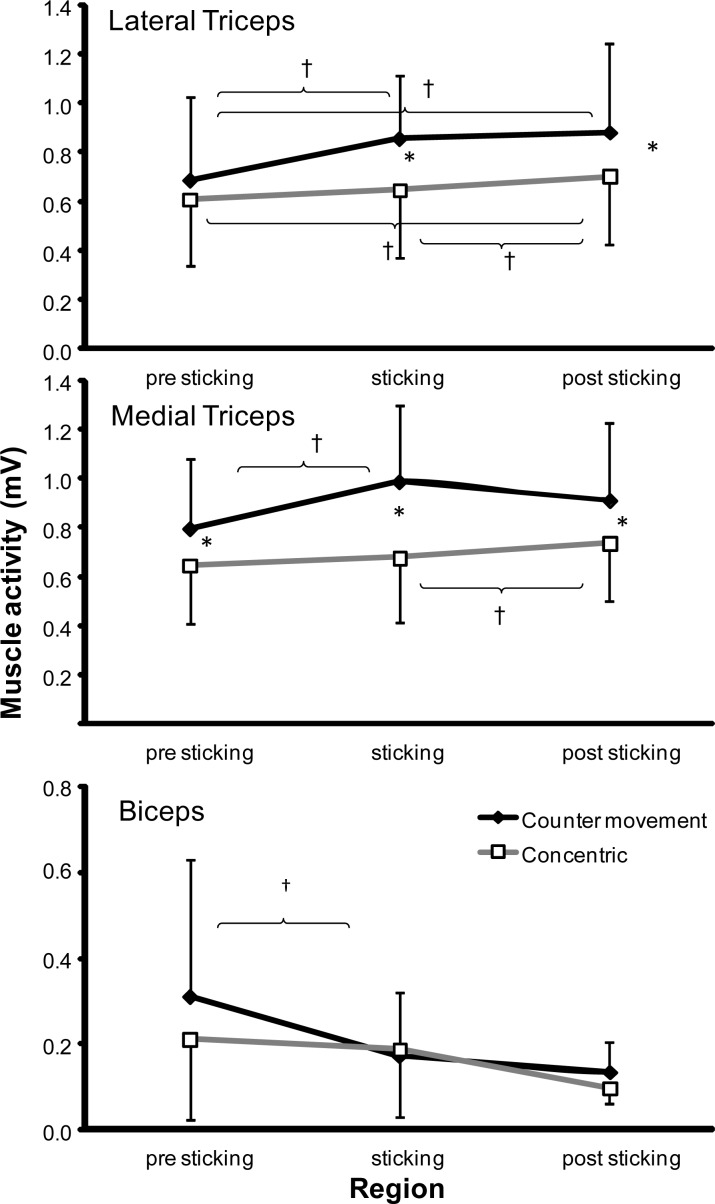
Mean muscle activities of the lateral and medial triceps and biceps muscles during the pre-, sticking and post-sticking region in the upward part during the counter movement and pure concentric bench press with their standard deviation. * indicates a significant difference in muscle activity at p ≤ *0.05 between the two conditions. † indicates a significant difference at p* ≤ *0.05 between these two regions for this condition*

**Figure 4:**
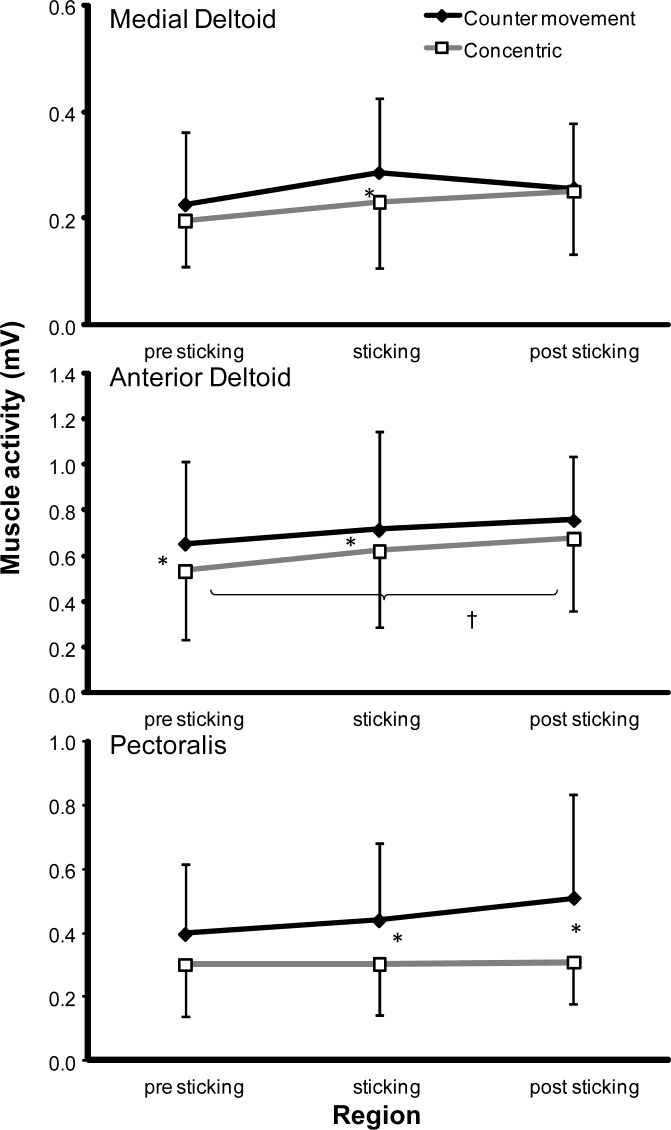
Mean muscle activities of the anterior and medial deltoid and major pectoralis muscles during the pre-, sticking and post-sticking region in the upward part during the counter movement and pure concentric bench press with their standard deviation. * indicates a significant difference in muscle activity at p ≤ *0.05 between the two conditions. † indicates a significant difference at p* ≤ *0.05 between these two regions for this condition*

Less total muscle activity was found in concentric than in counter movement condition for the lateral (*p* = 0.014) and medial triceps (*p* < 0.001), anterior deltoid (*p* = 0.02) and pectoralis muscles (*p* = 0.011), while no significant differences were found for the biceps (*p* = 0.378) and medial deltoid muscles (*p* = 0.108) (Figures 3 and 4). Post hoc comparisons revealed that for the lateral triceps and pectoralis the activity was significantly higher in CM, only in the sticking (*p* < 0.010) and post-sticking region (*p* < 0.036). The medial triceps activity was higher in every region (*p* < 0.020; Figures 3 and 4). The anterior deltoid activity was higher in CM, only in the pre-sticking (*p* = 0.011) and sticking region (*p* = 0.010) (Figure 4).

### Muscle activity between the three regions

Two-way Anova for repeated measures indicated a significant effect of the region factor on the lateral (p < 0.001) and medial triceps, (p = 0.036), biceps (p = 0.033), and anterior deltoid (*p* = 0.014) muscle activity while no significant difference in muscle activity between the regions was found for the pectoralis (p = 0.271) and medial deltoid muscles (p = 0.087) (Figures 3 and 4). Post hoc comparison revealed that the anterior deltoid, lateral and medial triceps activity increased from the pre-sticking region to the other two regions, but not from the sticking to post sticking region (Figures 3 and 4).

A different development of muscle activity (interaction: condition*region) between the two conditions was found only for the medial triceps; the medial triceps activity increased from the pre-sticking to the sticking region in the CM bench press, while in the CONC condition the muscle increased from the sticking to the post-sticking region (Figure 3). For the other muscles no significant differences in muscle activity development between the two conditions were found.

When comparing the regions per condition post hoc comparison showed that for the lateral triceps the pre-sticking activity was lower in the CM bench press compared to the other two regions, while in the CONC bench press the activity was higher in the post-sticking region compared to the other two regions (Figure 3). In the anterior deltoid, activity increased only significantly in the CONC bench press from the pre-sticking to the post sticking region (Figure 4).

## Discussion

In this study, the kinematics and muscle activity of six muscles in the ascending part of the 1-RM bench press between counter movement bench press and a pure concentric bench press were examined. In both conditions a sticking region occurred. However, the start of the sticking region was different between the two conditions. In addition in four of the six muscles, the muscle activity was higher in the CM bench press compared to the CONC one. However, the total impulse was the same for the two bench presses.

The occurrence of a sticking region in the CONC bench press, even though it started later than in the CM bench press, indicated that the proposed theory (van den Tillaar and Ettema, 2010) about the diminishing effect of potentiation, and a delayed muscle activation is unlikely the reason for the existence of a sticking region. The sticking region started when the barbell was at about 2 cm (CM) and 6 cm (CONC) from the sternum and ended at 11 and 13 cm from the sternum which was also found in earlier studies (Elliott et al., 1989; Wilson et al., 1991; McLaughlin and Madsen, 1984; Madsen and McLaughlin, 1984; Lander et al., 1985; Hamilton, 1995; Newton et al., 1997; van den Tillaar and Ettema, 2009; 2010). These findings indicate that around these heights the barbell is in a poor mechanical position to conduct maximal force by the participants (Van den Tillaar et al., 2012).

The participants lifted around 20 kg more with the CM bench press compared to the CONC lifts, which was also found in earlier studies (Wilson et al., 1991; Wilson et al., 1991). Even with this increased weight the initial acceleration was higher in the counter movement bench (Figure 2B) resulting in a higher first peak velocity (Figure 1). However, deceleration was also higher in these lifts, which may have been caused by diminishing potentiation of the contractile elements (Walshe et al., 1998). Furthermore, the difference in lifted weight was strongly associated with the significant increased muscle activity of the anterior deltoid and medial triceps muscles in the CM bench press (Figures 3 and 4). This was in accordance with the findings of Walshe et al. (1998) in squatting who concluded that higher active muscle state results in more force output in a counter movement than in a pure concentric movement. In addition, muscle potentiation may explain the higher performance in CM.

The total impulse was the same between the CM and CONC bench press indicating the participants had to conduct the same amount of force over time during the lift even when they lifted 20 kg less in the concentric condition. This indicates that in both conditions the participants conducted at their maximal potential in this set up.

In earlier studies, it was hypothesized that during the sticking region the lengths and mechanical advantages of the muscles involved were such that their capacity to exert force was reduced in this period (Madsen and McLaughlin, 1984; Elliott et al., 1989). In none of the prime movers (pectoralis, triceps and deltoid) the activity decreased from the pre- to the sticking region (Figures 3 and 4), indicating that muscle activity was not inhibited in the sticking region. Furthermore, only the medial and lateral triceps activity increases significantly in the CONC bench presses from the sticking to the post sticking region (Figure 3), demonstrating that the muscle activity level is not the limitation.

Significantly higher muscle activity was found in four of the six muscles during the ascending part of the lift with the CM bench press compared to the CONC bench press. This indicates that the muscle activity is not maximal when performing a concentric bench press. However, the muscle activity over the three regions behaved the same for almost all muscles. Only a different development was found for the medial triceps muscles, i.e. the muscle activity increased from the pre- to the sticking region in the CM condition, while in the CONC condition it increased from the sticking to the post-sticking region. The same trend was seen for the lateral triceps (but not significant). The reason for these differences is not clear, but they do not support the delayed muscle activity hypothesis (in association with diminishing potentiation).

This study had some limitations. Firstly, no joint angles of the elbow and shoulder were measured, which could give more information about the occurrence of the sticking region, i.e., if this sticking region is strictly dependent upon leverage and always occurs at the same angles for the participants. In future studies, this should be included before stating that the occurrence of the sticking region is the result of a poor mechanical position to conduct maximal force. Secondly the CM condition was always tested before the CONC condition, which could result in lower force and EMG outputs. However, in a study of Van den Tillaar et al. (2012), it was shown the force and EMG output was not affected negatively in later lifts after establishing first the 1-RM in the counter movement condition.

The kinematics in the ascending part of the 1-RM bench press between counter movement bench press and a pure concentric bench press revealed that both conditions have a sticking region. Together with the findings on muscle activity it was concluded that the diminishing effect of force potentiation, that occurs in CM bench presses, and delayed muscle activation unlikely explains the existence of the sticking region in a 1-RM bench press. Most likely, the sticking region is the result of a poor mechanical force position. Future studies in 1-RM bench press should be conducted that investigate this poor mechanical force production region by means of leverage changes during this region.

The results of the present study can help researchers, trainers and athletes in understanding sticking points and limitations of the muscles during pure concentric and counter movement bench pressing. We recommend that training should be targeted specifically at the sticking region: 3–16 cm vertically from the sternum during the lift (also in pure concentric lifts). This would help in increasing bench press performance since the sticking region is the weakest region during the lift (van den Tillaar and Ettema, 2009).
